# Androgen induced cellular proliferation, neurogenesis, and generation of GnRH3 neurons in the brain of mature female Mozambique tilapia

**DOI:** 10.1038/s41598-018-35303-9

**Published:** 2018-11-15

**Authors:** Yasuto Narita, Atsuhiro Tsutiya, Yui Nakano, Moe Ashitomi, Kenjiro Sato, Kohei Hosono, Toyoji Kaneko, Ruo-Dong Chen, Jay-Ron Lee, Yung-Che Tseng, Pung-Pung Hwang, Ritsuko Ohtani-Kaneko

**Affiliations:** 1 Department of Life Sciences, Toyo University, 1-1-1 Itakura, Oura, Gunma 374-0193 Japan; 2 Department of Aquatic Bioscience, Graduate School of Agricultural and Life Sciences, The University of Tokyo, 1-1-1 Yayoi, Bunkyo, Tokyo 113-8657 Japan; 3 Institute of Cellular and Organismic Biology, Academia Sinica, Nankang, Taipei City, Taiwan Republic of China

## Abstract

The neuroplastic mechanisms in the fish brain that underlie sex reversal remain unknown. Gonadotropin-releasing hormone 3 (GnRH3) neurons control male reproductive behaviours in Mozambique tilapia and show sexual dimorphism, with males having a greater number of GnRH3 neurons. Treatment with androgens such as 11-ketotestosterone (KT), but not 17β-estradiol, increases the number of GnRH3 neurons in mature females to a level similar to that observed in mature males. Compared with oestrogen, the effect of androgen on neurogenesis remains less clear. The present study examined the effects of 11-KT, a non-aromatizable androgen, on cellular proliferation, neurogenesis, generation of GnRH3 neurons and expression of cell cycle-related genes in mature females. The number of proliferating cell nuclear antigen-positive cells was increased by 11-KT. Simultaneous injection of bromodeoxyuridine and 11-KT significantly increased the number of newly-generated (newly-proliferated) neurons, but did not affect radial glial cells, and also resulted in newly-generated GnRH3 neurons. Transcriptome analysis showed that 11-KT modulates the expression of genes related to the cell cycle process. These findings suggest that tilapia could serve as a good animal model to elucidate the effects of androgen on adult neurogenesis and the mechanisms for sex reversal in the fish brain.

## Introduction

Sex reversal is a well-known phenomenon in fishes, with some naturally changing their phenotype during their lifetime and others switching sexes in response to environmental factors or hormone treatment. Fish which have undergone sex reversal have both gonads, but the reproductive behaviour of the sex they have reversed to^1^. Kobayashi *et al*. have reported that hormonal treatments induce heterotypical sexual behaviours in goldfish and carp^2^; however, they also retain and exhibit sexual behaviours of their original sex in response to key stimulus^3^. These findings indicate that fish tend to have some plasticity in their neural circuits.

Nile and Mozambique tilapias, commercially important fish species in aquaculture, are often subjected to artificial sex reversal during their early life stages because male fish grow faster than females. As such, androgen administration has been adopted for generating monosex (all male) tilapia^4,5^. Thorough investigation of the mechanisms underlying gonadal sex differentiation and reversal in tilapias have revealed that these processes are driven by the suppression of genes responsible for the production of one sex hormone and the activation of genes responsible for the production of the opposite sex hormone in the gonads^6–9^. However, it remains unclear how sex reversal takes place in the brain, though transcriptome analysis was recently applied to elucidate the mechanisms for the sex reversal in the brain^10,11^. The difficulty in elucidating this issue is partly because the specific neurons critically related to reproductive behaviours are not identified in sex reversal fish.

Gonadotropin-releasing hormones (GnRHs) are neuropeptides responsible for reproduction and other biological events in both vertebrates and non-vertebrates (for review, see^12–14^). Most teleost fishes (including tilapias) have three GnRH subtypes (GnRH1, GnRH2, and GnRH3). Using Nile tilapia, Ogawa *et al*.^15^ established that GnRH3 is a potent neuromodulator for male sexual behaviours such as nesting and aggression^15^. They found that injection of anti-GnRH antisera into the third ventricle of the male fish brain (intraventricular GnRH-immunoneutralization) resulted in significantly decreased nest-building ability, nest size, and aggressive behaviour^15^. Our previous study^16^ that focused on GnRH3 neurons in the tilapia brain, revealed that GnRH3 neurons are localized to the terminal nerve (TN, terminal ganglion) and that mature males have significantly more GnRH3 neurons than mature females. Additionally, androgens such as 11-ketotestosterone (11-KT, fish specific androgen) and methyltestosterone (potent synthetic fish androgen), but not oestrogen, could annul the difference in the number of GnRH3 neurons between the sexes^16^. In other words, the number of GnRH3 neurons in androgen-treated females were increased to a level similar to mature males, revealing that the brains of androgen-treated females were sexually reversed in terms of GnRH3 neurons. Furthermore, androgen injection produced male-specific nest-building behaviour in about 70% of mature females, within 2 weeks after the 11-KT injection^16^. Taken together, these studies indicate that GnRH3 neurons are important in regulating reproductive behaviours of males and are also involved in sex reversal of the brain in females. However, it is still uncertain how 11-KT increases GnRH3 neurons in the female brain. Hence, it is especially important to answer whether or not 11-KT increases GnRH3 neurons via adult neurogenesis.

Numerous studies using various vertebrate species have shown that oestrogen can induce neuronal proliferation in adults^17^ (and see^18–20^ for reviews on the positive effects of oestrogen on adult neurogenesis in rodents). In contrast, some studies have reported inhibitory effects of androgens on adult neurogenesis in the mouse brain and rhesus macaque hippocampus^21,22^, whereas other studies on song birds have shown positive effects of androgen on adult neurogenesis^23–26^. However, since testosterone is converted to estradiol by aromatase in the brain, it is difficult to determine whether testosterone itself has effects on adult neurogenesis. Therefore, it is of great importance to know whether androgens themselves can induce adult neurogenesis and generate new neurons with identified functions like GnRH neurons. A non-aromatizable androgen, 11-KT is the most potent natural androgen in teleosts^27,28^ and is a non-aromatizable androgen. This property of 11-KT makes it easier to address the question whether the androgen itself, but not oestrogen converted from androgen, is responsible for stimulating adult neurogenesis in androgen-treated fish.

The present study thus aimed to investigate two questions by using mature female Mozambique tilapia. The first question is whether 11-KT can cause cellular proliferation, adult neurogenesis, and altered gene expressions related to cell cycle in the brain and the second question is whether 11-KT can increase GnRH3 neurons, via adult neurogenesis, during masculinization in the females.

## Results

### 11-KT-induces increase in proliferating cells (PCNA-positive) in the periventricular regions of mature females

Our previous study demonstrated that GnRH3 neurons were sexually dimorphic in the Mozambique tilapia; males have a greater number of GnRH3 neurons in the TN and treatment with androgens, such as 11-KT and methyltestosterone, increases the number of GnRH3 neurons in mature females to a level similar to mature males^16^. In order to examine whether 11-KT affects cellular proliferation in this brain region, we compared the number of proliferating cell nuclear antigen (PCNA)-positive cells between control and 11-KT-injected females.

**Figure 1:**
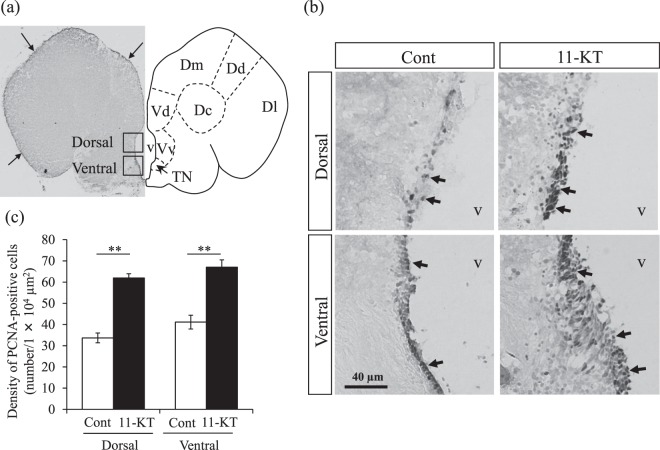
PCNA immunostaining in the female tilapia brain. (**a**) Left: Representative frontal section of the female tilapia brain crossing the terminal nerve (TN) immunostained with PCNA antibody. The two inserted squares correspond to regions in the dorsal and ventral areas used for counting cells displaying a PCNA-positive nucleus (each area, 140 µm × 140 µm) around the ventricle. Arrows indicate PCNA-positive cells. Right: Atlas of the brain corresponding to the left image. Dm = medial zone of dorsal telencephalic area; Dd = dorsal zone of dorsal telencephalic area; Dl = lateral zone of dorsal telencephalic area; Vd = dorsal nucleus of ventral telencephalic area; Vv, = ventral nucleus of ventral telencephalic area; v = ventricle. (**b**) Representative image of PCNA-positive cells in the dorsal and ventral areas around the ventricle at the level of the TN in 11-KT-treated and control (Cont) female tilapia. Arrows indicate PCNA-positive nuclei. (**c**) Density of PCNA-positive cells in control and 11-KT-treated females in the dorsal and ventral areas around the ventricle. **Indicates *p* < 0. 01 (unpaired Student’s *t*-test). Data are expressed as mean ± SEM.

Immunohistochemistry using anti-PCNA antibody revealed that PCNA-positive nuclei were present along the outer surface of the brain (arrows in Fig. 1a) and around the ventricle (periventricular regions, arrows in Fig. 1b). Since the TN is located near the ventricle^16,29^, we evaluated the effect of 11-KT on cellular proliferation around the ventricle. As shown in Fig. 1b, PCNA-positive cells were observed in the dorsal and ventral periventricular regions of both control and 11-KT-injected females; interestingly, PCNA-positive cells were much more densely detected in 11-KT-injected females than in controls.

Bilateral counts of PCNA-positive cells in the dorsal and ventral periventricular regions (indicated in black squares in Fig. 1a), measured in the frontal section cutting through the TN, revealed that the mean density of PCNA-positive cells was significantly higher in 11-KT injected females than in controls (*p* < 0.01, Student *t*-test, Fig. 1c). Thus, these studies demonstrated that administration of 11-KT significantly increased cellular proliferation along the ventricle.

### Androgen increased generation of neurons, but not radial glial cells around the ventricle

In order to determine the phenotype of the newly proliferated cells in the brain area around the TN, we combined BrdU-tracing with immunostaining against a neuron marker, Hu, or a radial glial marker, glial fibrillary acidic protein (GFAP). BrdU-tracing experiments were performed by simultaneous injections of BrdU either with 11-KT or with the vehicle (oil).

**Figure 2:**
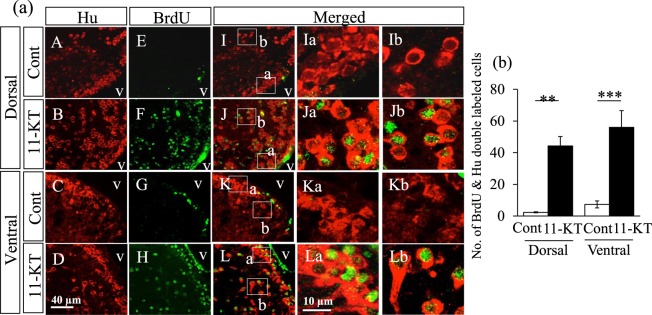
Representative images obtained from double immunohistochemistry with BrdU and Hu antibodies. (**a**) Hu-positive cells (red) observed in dorsal and ventral areas around the ventricle in control (cont) and 11-KT-treated (11-KT) females (A,B,C,D). Although few BrdU-labelled nuclei (green) were detected in control animals (E,G), BrdU-labelled nuclei were frequently observed in 11-KT-treated females (F,H). I,J,K and L show merged images. Ia,b,Ja,b,Ka,b and La,b show enlarged images of the areas inset in I,J,K, and L, respectively; v = ventricle. (**b**) The number of Hu-positive cells with a BrdU-labelled nucleus. White and black bars indicate the numbers of double-labelled cells in control (Cont) and 11-KT-treated (11-KT) females. **Indicates *p* < 0. 01; ***Indicates *p* < 0. 001 (unpaired Student’s *t*-test). Data are expressed as mean ± SEM.

Female fish simultaneously injected with BrdU and 11-KT (or vehicle) had Hu-positive neurons in the dorsal and ventral parts of the brain (red cells in Fig. 2a-A–D and I–L). Only a few BrdU-positive nuclei were detected in controls (green nuclei in Fig. 2a-E and G); however, numerous BrdU-positive nuclei were observed along the ventricle and in substantial areas in the dorsal and ventral parts of the brain in 11-KT-injected females (green nuclei in Fig. 2a-F and H). Similarly, 11-KT-treated female fish displayed many Hu-positive cells with a BrdU-labelled nucleus (newly-generated/proliferated neurons) along the ventricle and in substantial areas of the dorsal and ventral parts of the brain (red cells with green nuclei in Fig. 2a-J and L). In contrast, the double-labelled cells were scarcely detected in control females (Fig. 2a-I and K). Higher magnification images further confirmed our observation that double-labelled cells (BrdU- and Hu-positive cells) were scarcely observed in control females along the ventricle (Fig. 2a-Ia and Ka) or substantial areas of the brain in the controls (Fig. 2a-Ib and Kb), while 11-KT-treated females displayed multiple double-labelled cells in these areas (Fig. 2a-Ja, Jb, La, and Lb).

As shown in Fig. 2b, the number of newly-generated neurons (Hu-positive cells with a BrdU-labelled nucleus) in the dorsal and ventral periventricular regions, in a frontal section cutting through the TN, were markedly increased in 11-KT-injected females, compared with controls (*p* < 0.01 or *p* < 0.001; controls *vs* 11-KT-treated females, unpaired Student’s *t*-test). These results demonstrate that 11-KT increased adult neurogenesis in the dorsal and ventral regions along the ventricle in females.

**Figure 3:**
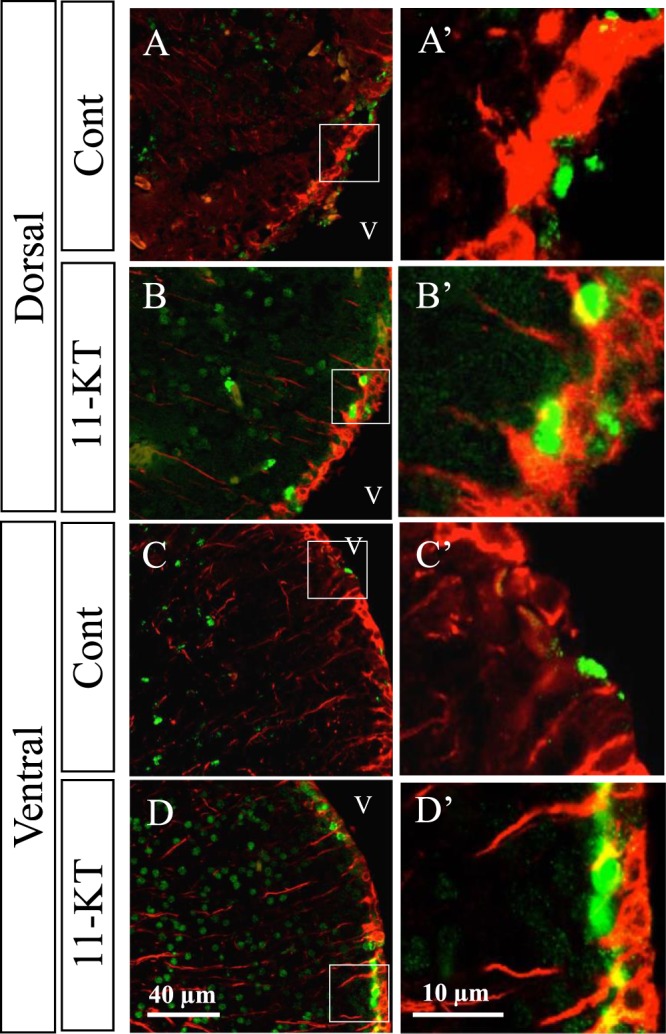
Representative merged images of sections double-stained with BrdU and GFAP antibodies. GFAP-positive cells (red) visualized on the inner surface of the ventricle, with long processes in the dorsal and ventral areas around the ventricle in control (Cont) and 11-KT-treated (11-KT) females. (**A**,**B**) dorsal and ventral areas, respectively, of control animals. (**C**,**D**) dorsal and ventral areas, respectively, of 11-KT-treated females. A’,B’,C’ and D’ show enlarged views of inset squares in A,B,C, and D, respectively; v = ventricle.

GFAP-immunoreactive ventricular radial glial cells in teleost fish are considered as astrocytic subpopulations and neuronal progenitors in mammals^30^. We examined whether 11-KT could affect the proliferation of radial glial cells around the ventricle, in the frontal section cutting through the TN, in female tilapia. Figure 3 shows representative images of immunostaining with antibodies against GFAP and BrdU. We found many GFAP-positive cells in the dorsal and ventral areas around the ventricle in both control and 11-KT-treated females (red cells in Fig. 3); however, no GFAP-positive cells with a BrdU-labelled nucleus were observed (Fig. 3A’–D’). These findings indicated that 11-KT did not increase the proliferation of ventricular radial glial cells around the TN in the female brain.

### 11-KT-generated GnRH3 neurons in female tilapia

**Figure 4:**
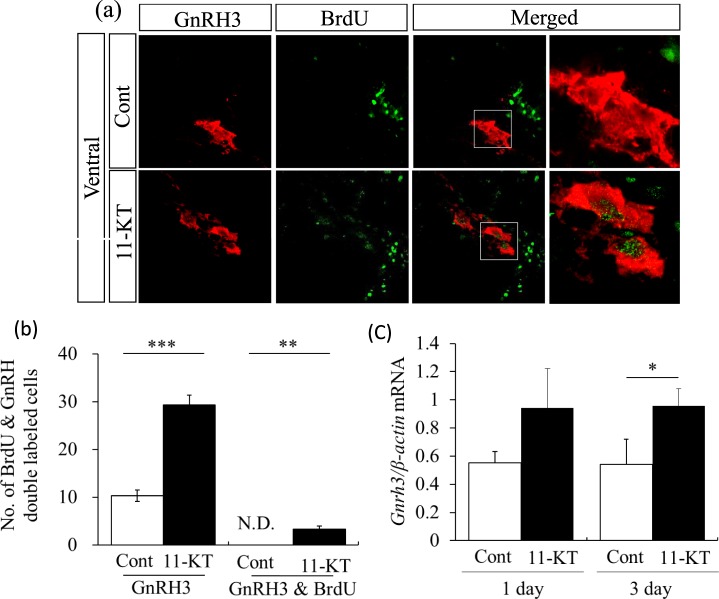
Representative images of sections double-stained with BrdU and GnRH3 antibodies. (**a**) GnRH3 neurons (red), BrdU-labelling nuclei (green) and merged images in control (Cont) and 11-KT treated (11-KT) females. GnRH3 neurons with BrdU-labelling in the nucleus were observed in 11-KT treated females, but not in controls. The rightmost column shows enlarged views of inserted squares in the second from the right column. (**b**) Number of GnRH3 neurons in control (Cont, white column) and 11-KT-treated (11-KT, black column) females (left) three days after 11-KT injection. Number of double labelled cells (GnRH & BrdU) in control (Cont, white column) and 11-KT treated (11-KT, black column) animals (right); N. D. = not detected. (**c**) Relative expression levels of GnRH3 mRNA in control (Cont, white column) and 11-KT treated (11-KT, black column) females one day (left) and three days (right) after 11-KT injection. **Indicates p < 0.01; ***Indicates p < 0.001 (unpaired Student’s t-test). Data are expressed as mean ± SEM.

Further examination of the effect of 11-KT on GnRH3 neurons revealed that no control females exhibited GnRH3-positive neurons (red) with a BrdU-labelled nucleus (Fig. 4a, “Cont”). In contrast, GnRH3 neurons (red) with a BrdU-positive nucleus (green) were found in female tilapia injected with 11-KT (Fig. 4a). These findings indicated that 11-KT treatment could induce newly-generated GnRH3 neurons in the female tilapia brain.

As shown in Fig. 4b, the mean number of GnRH3-positive neurons was significantly increased in 11-KT-treated fish, compared with control females at three days after 11-KT injection (p < 0.001, Cont *vs* 11-KT in the left side columns in Fig. 4b). GnRH3 neurons with a BrdU-labelled nucleus were detected in 11-KT-treated females (Fig. 4b), but not in controls.

Our transcriptome analysis showed that the expression level of GnRH3 mRNA was increased by 11-KT one day after treatment (fold change = 1.135, Supplementary Table 3). As shown in Fig. 4c, quantitative real-time reverse transcription polymerase chain reaction (qRT-PCR) analysis showed that the expression of GnRH3 mRNA was increased significantly at 3 days after 11-KT injection (*p* < 0.05, student *t*-test), but not significantly at day 1.

### 11-KT-induced changes in cell cycle-related gene expressions

**Figure 5:**
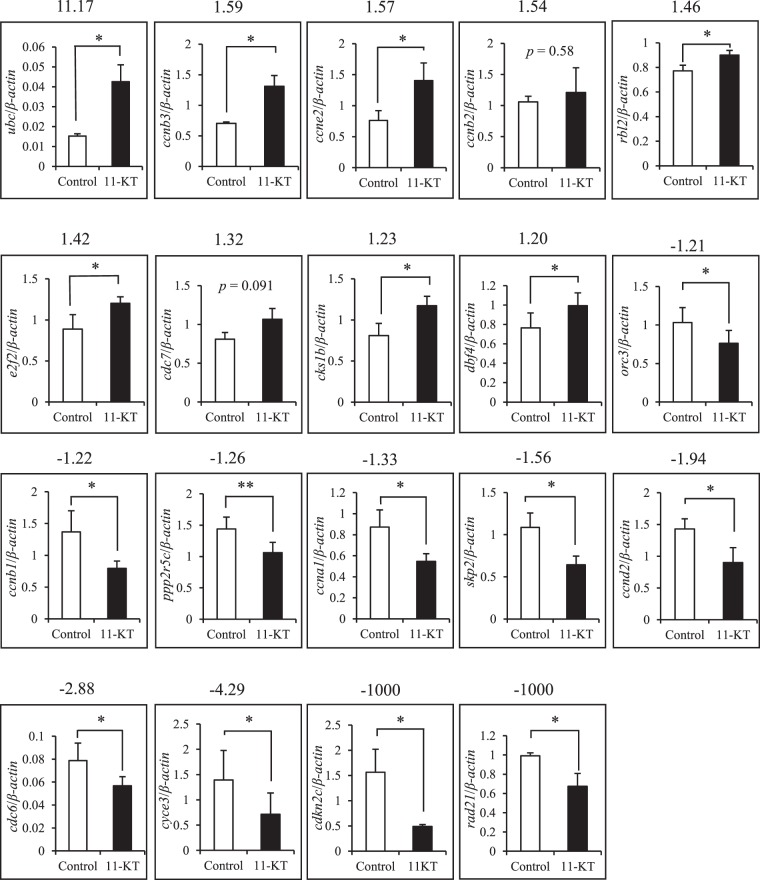
Changes in expressions of genes related to cell cycle after 11-KT treatment. Expressions of 19 genes listed in Table 1 were examined by real-time RT-qPCR analysis and compared between control (white column) and 11-KT treated (11-KT, black column) females. The numeric values on the top of each graph indicates the fold change as revealed by the transcriptome analysis. *Indicates *p* < 0. 05, **Indicates *p* < 0.01 (unpaired Student’s *t*-test). Data are expressed as mean ± SEM.

**Figure 6:**
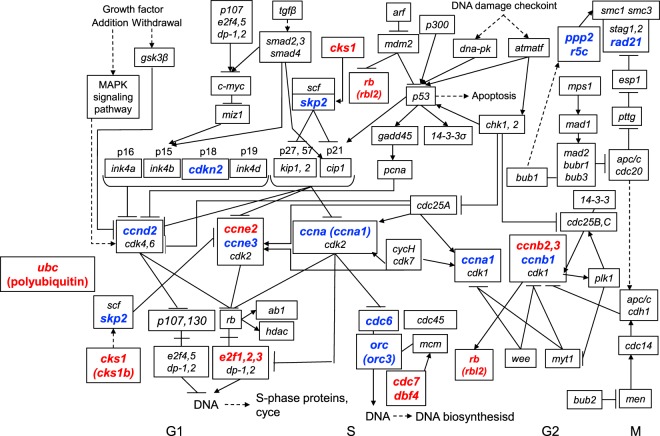
Cell cycle regulation pathway and the genes whose expressions were altered by 11-KT. The figure was slightly modified from ‘Cell cycle’ pathway (map04110, KEGG). Genes whose expressions were increased (red, fold change >1. 2) or decreased (blue, fold change < -1. 2) by 11-KT treatment were marked in the figure. *Ubc* coding polyubiquitin-C precursor regulates each phase of the cell cycle through the degradation of cyclins.

Transcriptome analysis was conducted to compare changes in gene expression in the brain region around the TN between control and 11-KT-treated female groups. Transcriptome profiling revealed that 1122 genes (among a total number of 6851 assembled unigenes) were identified as having a 1.2-fold change due to treatment with 11-KT; 641 genes were upregulated by 11-KT treatment, while 481 genes were downregulated. To evaluate the androgen effects on cellular proliferation, we focused on transcripts that were clustered within “Cyclins and Cell Cycle Regulation” pathway in PathCards and “Cell Cycle” pathway according to the Kyoto Encyclopedia of Genes and Genomes (KEGG). It was found that 103 genes were identified based on the transcriptome profiling. As shown in Table 1, using a plus 1.2-fold change as the threshold, we found that 9 transcripts were up-regulated by 11-KT, namely, polyubiquitin C (*ubc*), cyclin B3 (*ccnb3*), cyclin E2 (*ccne2*), cyclin B2 (*ccnb2*), retinoblastoma-like protein 2 (*rbl2*), E2F transcription factor 2 (*e2f2*), cell division control protein 7 (*cdc7*), cyclin-dependent kinases regulatory subunit 1 (*cks1b*), and activator of S phase kinase (*ask* or *dbf4*). Similarly, using a minus 1.2-fold change as the threshold, the following 10 genes were down-regulated by 11-KT, namely, origin recognition complex subunit 3 (*orc3*), cyclin B1 (*ccnb1*), protein phosphatase 2 regulatory subunit B’gamma (*ppp2r5c*), cyclin A1 (*ccna1*), S-phase kinase-associated protein 2 (*skp2*), cyclin D2 (*ccnd2*), cell division control protein 7 (*cdc6*), G1/S-specific cyclin E3 (*cyce3*), cyclin-dependent kinase N2C (*cdkn2c*), and cohesion complex subunit SCC1 (*rad21*). Our qRT-PCR analysis that examined expression of these 19 genes (Supplementary Table S2) revealed that levels, except *ccnb2* and *cdc7*, were significantly affected by 11-KT in the female brain (Fig. 5). In addition, the tendency of 11-KT-induced effects on *ccnb2* and *cdc7*, as shown by qRT-PCR assay, was consistent with the results obtained from our transcriptome analysis. As shown in Fig. 6 and Table 1, these 19 genes are inferred to play key roles in the cell cycle regulation as positive or negative regulators, mostly in G1 and S phases.

**Table 1:** Cell cycle related gene expressions which were affected by 11-KT.

Gene Symbol		Gene Name	Accession No.	Fold Change (11-KT/Cont)	Function
*ubc*		polyubiquitin	XM_019365938	11.17	ubiquitin gene, Ubiquitination has been associated with protein degradation, DNA repair, cell cycle regulation, kinase modification, endocytosis, and regulation of other cell signaling pathways.
*ccnb3*		cyclin B3	XM_005458602 XM_003453924	1.586	Regulator of cyclin dependent kinase
*ccne2*		cyclin E2	XM_005472790	1.567	Regulator of cyclin dependent kinase
*ccnb2*		cyclin B2	XM_005457408 XM_003452594	1.541	Regulator of cyclin dependent kinase.The B-type cyclins, B1 and B2, associate with p34cdc2 and are essential components of the cell cycle regulatory machinery
*rbl2*		retinoblastoma-like protein 2	XM_019360388 XM_019360390 XM_019360401 XM_005447651 XM_019360394	1.464	Key regulator of entry into cell division
*e2f2*		transcription factor E2F2	XM_003453243 XM_005477160	1.422	The E2F family plays a crucial role in the control of cell cycle and action of tumor suppressor proteins and is also a target of the transforming proteins of small DNA tumor viruses.
*cdc7*		cell division control protein 7	XM_005476033 XM_005476034	1.321	ATP binding, kinase activity, metal ion binding, protein binding, protein kinase activity, protein serine/threonine kinase activity
*cks1b*		CDC28 protein kinase regulatory subunit 1B	XM_003452448	1.234	cyclin-dependent protein serine/threonine kinase activator activity, histone binding, protein binding, protein kinase binding, ubiquitin binding
*dbf4*	*ask*	activator of S phase kinase	XM_003450951 XM_005472703 XM_019364488	1.204	Regulatory subunit for CDC7 which activates its kinase activity thereby playing a central role in DNA replication and cell proliferation.
*orc3*		origin recognition complex subunit 3	XM_003453228	−1.211	DNA replication origin binding, protein binding
*ccnb1*		cyclin B1	XM_003440084	−1.223	Regulator of cyclin dependent kinase.The B-type cyclins, B1 and B2, associate with p34cdc2 and are essential components of the cell cycle regulatory machinery
*ppp2r5c*		protein phosphatase 2 regulatory subunit B’gamma	XM_003440783 XM_005449702 XM_005449704	−1.257	Phosphatase 2 A regulatory subunit B family. It is implicated in the negative control of cell growth and division.
*ccna1*		cyclin A1	XM_003458268	−1.335	Regulator of cyclin dependent kinase
*skp2*	*fbxl1*	F-box and leucine-rich repeat protein 1 (S-phase kinase-associated protein 2)	XM_005449432	−1.557	SKP2 has been implicated in double negative feedback loops with both p21 and p27, that control cell cycle entry and G1/S transition
*ccnd2*		cyclin D2	XM_003442521	−1.941	Regulator of cyclin dependent kinase
*cdc6*		cell division control protein 6	XM_003454088	−2.878	ATP binding, kinase binding, nucleotide binding, protein binding
*cyce3*		cyclin-E3	XM_019351010 XM_019351007 XM_019351009 XM_005452832	−4.291	Regulator of cyclin dependent kinase
*cdkn2c*		cyclin-dependent kinase inhibitor 2 C	XM_019351452 XM_019351453 XM_019351454	−1000	cyclin-dependent protein serine/threonine kinase inhibitor activity
*rad21*	*scc1,mcd1*	cohesin complex componet	XM_003447160	−1000	The RAD21 gene provides instructions for making a protein that is involved in regulating the structure and organization of chromosomes during cell division.

The table provides a description that includes the gene symbol, name and function for each gene affected by 11-KT treatment. It also gives the fold change (11-KT/control) for each gene that was affected by 11-KT treatment.

## Discussion

This study revealed the promotive activity of androgens on cellular proliferation, adult neurogenesis, and the production of GnRH3 neurons, partly *via* adult neurogenesis, in the female tilapia brain. Since testosterone, the major androgen in mammals and birds^31^, can be converted to estradiol by aromatase, it is difficult to discern the effects of testosterone from that of estradiol in these animals. Importantly, 11-KT is a non-aromatizable androgen and the most potent natural androgen in teleosts^27,28^ and helped identify effects of androgen on mature female tilapia in this study. A previous study by our group has shown that 11-KT and methyltestosterone, but not estradiol, increased the number of GnRH3 neurons in female tilapia, suggesting the promotive role of androgen in the proliferation and/or differentiation of GnRH3 neurons^16^.

Immunostaining with PCNA antibody revealed that 11-KT treatment had a positive effect on cellular proliferation in the brain. BrdU-tracing combined with Hu-immunostaining suggested that 11-KT induced adult neurogenesis in mature females. On the other hand, BrdU-tracing combined with GFAP-immunostaining indicated that 11-KT treatment did not stimulate the production of newly-generated ventricular radial glial cells, which are regarded as progenitor cells in the adult fish brain^30^. Although they are mostly transient in developing mammals, radial glial cells are maintained in the adult central nervous system of fish, and serve as guides for migrating neurons as well as the major source of neural and glial precursor cells [for review, see^32^]. Perez *et al*.^33^ have reported that PCNA-positive cells correspond to GFAP-positive radial glial cells in fish^33^. These radial glial cells in fish are reported to express steroid receptors including androgen receptors, and are directly targeted by steroids^34,35^. Thus, combined with our present results, it is indicated that 11-KT may have induced mitosis of radial glia cells (PCNA-positive cells) which produced newly-generated neurons (Hu- and BrdU-double labelled cells) but not newly-generated radial glial cells (GFAP- and BrdU-double labelled cells).

In addition, based on BrdU-tracing combined with GnRH3-immunostaining, 11-KT treatment produced newly-proliferated GnRH3 neurons. These results revealed that some of the 11-KT increased GnRH3 neurons in the female brain^16^ are probably generated *via* adult neurogenesis. GnRH3 neurons can be generated through one of three possible mechanisms: (1) proliferation from progenitor cells and differentiation after cell division, (2) differentiation from pre-existing cells without proliferation, or (3) the combination of both. Our data support the idea that, at least, some of the new GnRH3 neurons could have resulted from adult neurogenesis. However, since the GnRH3- and BrdU-double labelled cells were only a small part of GnRH3 neurons (Fig. 4b), it is a possible scenario that differentiation into GnRH3 neurons from pre-existing cells may be a major contributor. As a next step, we will be examining the precise pathways possibly involved in the neurogenesis and differentiation of GnRH3 neurons induced by 11-KT in mature females. Because it is difficult to investigate the direct role of androgen and pathways *in vivo*, a pharmacological approach using brain slices including the terminal ganglion area will be applied.

The generation of GnRH neurons during development has been well studied in vertebrates, GnRH (or luteinizing hormone-releasing hormone) neurons originate in the medial olfactory placode of the developing rodent nose and migrate into the septal-preoptic area and hypothalamus^36–38^. In the sockeye salmon, Parhar *et al*.^29,39^ established that GnRH3 neurons originated in the medial olfactory placode and migrated into the basal forebrain during development. Using the *gnrh3*-GFP transgenic medaka, Okubo *et al*.^40^. visualized the migration pathway of GnRH neurons including GnRH3 neurons during development. On the contrary, the generation of GnRH neurons, including GnRH3 neurons, in adult animals remains unclear. In the Tg (GnRH3:EGFP) zebrafish line, after specific ablation of GnRH3 neurons in the olfactory region, GnRH3 neurons were regenerated only when the ablation was conducted 2 days after fertilization^41^. Interestingly, bilateral GnRH3 soma ablation at 4 or 6 days after fertilization resulted in lack of GnRH3 neurons and arrested oocytes in adults. Additionally, in female zebrafish without hormonal treatments, the only source of GnRH3 neurons in the olfactory region in embryos^41^. On the other hand, new-born GnRH1 neurons, with GnRH1-immunoreactivity and BrdU labelling in the nuclei, were detected in the adult ring dove forebrain after brain lesion and the recruitment of new GnRH1-immunoreactive neurons is sensitive to the reproductive stage of the birds^42^. Cortés-Campos *et al*.^43^ demonstrated that adult zebrafish-derived hypothalamic neurospheres generate GnRH neurons, including GnRH3 ones. They also demonstrated that the number of GnRH neurons increased following exposure to testosterone or GnRH3. In accordance with the latter two studies, the present result supports the idea that GnRH neurons in some vertebrates can be generated in certain conditions, like lesions or hormonal treatments such as androgen exposure.

Tilapia have distinct sexual dimorphism in the number of GnRH3 neurons; mature males have more GnRH3 neurons than mature females^16^. Multiple studies have reported sex differences in GnRH3 neurons or *gnrh3* expression. For example, female half spotted goby had more and larger GnRH cells in the TN^44^. Similarly, in medaka, the expression of *gnrh3* was slightly higher in females than males, although no sex differences in the total area of *gnrh3* expression were detected^45^. In tilapia, other than courtship behaviours, sexual reproductive behaviours were clearly different between the sexes; males exhibited nest building and aggressive behaviours, whereas in females maternal oral egg incubation was observed. It is reported that GnRH3 is a potent neuromodulator of male reproductive behaviours in tilapia^15^. Thus, the strong neuromodulatory role of GnRH3 neurons on the male-specific reproductive behaviours is related to the large population of GnRH3 neurons in male tilapia and the promotive effect of 11-KT on them. In addition, we confirmed that the expression level of GnRH3 mRNA was also increased by 11-KT treatment.

Among the gonadal hormones, estradiol has been recognized as a major modulator of adult neurogenesis due to its capacity to facilitate both survivability and proliferation of neurons in mammals and birds^17–20^. In addition, aromatase is localized in radial glial cells in the adult teleost^30,46^, [see^47^ for review] and oestrogens have been suggested to play significant roles in the brains of these fishes. However, actions of oestrogen in fish still remains controversial. Diotel *et al*.^48^ established the inhibitory action of estradiol on cell neurogenesis, and migration at the olfactory bulbs/telencephalon junction and in the mediobasal hypothalamus of the adult zebrafish brain. Blocking the aromatase activity with 1,4, 6-androstatriene-3,17-dione or oestrogen receptors with ICI 182,780 increased the number of proliferative cells, suggesting an inhibitory role for estrogen in the proliferation in the fish brain. On the contrary, Lin *et al*.^49^ showed that estradiol treatment in the early brain development enhanced cellular proliferation and gene expressions of aromatase and radial glial cell marker (*Blbp*) in black porgy indicating a stimulatory effect of estradiol on the proliferation and development of radial glial cells in the fish brain^49^. Research studies reporting the androgen-induced neurogenesis in vertebrates are much less compared to those that reporting neurogenesis induced by oestrogen. Most of these studies focused on the song control centre (HVC) of the brain in song birds and reported testosterone-induced enlargement of the HVC due to neurogenesis, angiogenesis and neuronal survivability increased by the hormone^23–26,50–53^. In most of these studies, however, the effect of oestrogen aromatized from testosterone cannot be excluded. In mammals, there is a non-aromatizable androgen, 5α-dihydrotestosterone (5α-DHT). In addition, androgenic mechanisms of sexual differentiation of the central nervous system and behavior have been well documented in mammals during development, especially by using genetically modified mice or mutants [see for reviews^54,55^]. In contrast with mammals, sex reversal can occur in many species of mature fishes, whose brain holds plenty of neural progenitor cells during adulthood. However, little information is available on whether androgen may induce sex reversal of the brain, or whether it may induce adult neurogenesis in the fish brain. We provide the first evidence that androgen can positively affect adult neurogenesis in the fish brain. This may partly constitute the mechanisms by which the sex reversal of the brain occurs under androgen exposure.

The mechanistic machinery for 11-KT to induce neurogenesis in the mature female tilapia brain remains unsolved. Bases on our transcriptome data, we suggest that among the *Arα, Arβ, Erα, and Erβ* genes, *Arβ* gene expression is indeed upregulated by 11-KT in females (Supplementary Table S3). Furthermore, present transcriptome profiling with qRT-PCR analysis revealed that 11-KT affected the expressions of genes related to the cell-cycle pathway in the region around the TN (Figs 5 and 6). We found that among the members of the cyclin family proteins which control progression of the cell cycle, transcript expressions of *ccnb3, ccnb2* and *ccne2* were increased, while *ccnb1, ccna1, ccnd2*, and *cyce3* were decreased, by 11-KT treatment. Importantly, each cyclin homolog is differentially expressed at various cell cycle phases. For example, it has been reported that cyclin B1 functions during the M phase, while cyclin B3 is a mitotic cyclin that promotes the metaphase-anaphase transition^56^. E-type cyclins function in driving cell cycle progression^57^, whereas cyclin A1 functions mainly in the G1/S cell cycle progression^58^. In addition to cyclins, expression of other genes known as cell cycle regulators, was positively or negatively affected by 11-KT. For example, gene expressions of *rbl2* and *e2f2*, crucial regulators of cell division^59^, were stimulated by 11-KT treatment. In contrast, the expressions of *skp2* and *cdkn2c*, which are reported to control CDK activity^60,61^ were depressed. The large increment of *ubc* mRNA induced by 11-KT (Fig. 6) supports the importance of the ubiquitin-proteasome degradation system’s involvement in cell cycle regulation, similar to as seen in rodents^62^. Since most proteins translated from these genes positively or negatively regulate each other through phosphorylation or degradation during the cell cycle, it is difficult to decide from these data whether 11-KT has a positive or negative effect on cell cycle. In conclusion, these results obtained from the transcriptome analysis and real-time qRT-PCR analysis support the idea that 11-KT can influence cellular proliferation and fit the results from PCNA-staining and BrdU-tracing experiments in the female tilapia brain.

## Methods

### Animals

Experiments were conducted according to the principles and procedures approved by the Institutional Animal Care and Use Committees of Toyo University and Academia Sinica. Mozambique tilapia (*Oreochromis mossambicus*) were maintained at 25 °C. The total number of tilapia used was 115.

### Comparison of the number of proliferating (PCNA-positive) cells around the TN between control and 11-KT-treated females

#### 11-KT injection

Sexually mature female Mozambique tilapia of almost equal size (n = 6; approximately 30 g in body weight (BW) and 15 cm in length) were used. 11-KT (Cosmo Bio, Japan) was dissolved in sesame oil at a concentration of 1.0 mg/ml. Three mature females (11-KT-treatment) were intraperitoneally injected with 11-KT at 5 µg/g BW, and three mature females (controls) were injected with sesame oil alone. After the injections, fish were housed individually in tanks (50 L glass tanks) for 7 days.

#### Brain fixation

Seven days after the injection, animals were anesthetized with 0.2% 3-aminobenzoic acid ethyl ester (MS222, Sigma, St. Louis, MO) and transcardially perfused with saline, followed by 4% paraformaldehyde in 0.1 M phosphate buffer (PB, pH 7.3). Brains were then removed and post-fixed in the same fixative overnight.

#### Brain sectioning

Fixed brains were washed in 0.1 M phosphate-buffered saline (PBS), and then immersed in 0.1 M PB containing 20% sucrose overnight. Serial frontal sections (20 μm thickness) were cut on a cryostat (CM-3050-S, Leica Microsystems, Wetzlar, Germany), thaw-mounted onto MAS-coated glass slides (Matsunami Glass, Osaka, Japan) in three parallel series, and stored at −20 °C until further use.

#### Immunohistochemistry for localization of GnRH3 neurons in the TN

Two of the three series of brain sections from each fish were used for PCNA immunohistochemistry, while the other series was used for GnRH3 labelling to confirm the location of the TN. Immunohistochemistry with an anti-GnRH3 antibody was performed using a custom-made primary anti-GnRH3 antibody (produced by Protein Purify, Isezaki, Japan) and Alexa 555-labelled goat anti-rabbit IgG (ThermoFisher Scientific LTD)^16^. GnRH3-positive neurons were observed under a fluorescence microscope (Axio Imager A1, Zeiss, Jena, Germany) and sections with the highest number of GnRH3-positive cells were identified.

#### Proliferating cell nuclear antigen (PCNA) immunohistochemistry and cell counts

For PCNA immunohistochemistry, we chose the sections immediately adjacent to the one identified as having the highest number of GnRH3 neurons. These sections were incubated in methanol containing 0.3% hydrogen peroxide to block endogenous peroxidase activity. Next, sections were immunohistochemically stained with mouse monoclonal anti-PCNA antibody (1:3000, P8825, Sigma-Aldrich) diluted in 0.1 M PBS containing 0.03% Triton X-100 and 10% Block Ace and with biotinylated goat anti-mouse IgG (1:500, Vector Laboratories, Burlingame, CA, USA). After incubation with an avidin-biotin-peroxidase-complex (ABC Method kit, Vector Laboratories), the sections were incubated with 0.02% solution of 3, 3′-diaminobenzidine (Wako Pure Chemical Industries) in 0.175 M sodium acetate buffer containing 0.125% nickel chloride (Wako Pure Chemical Industries, Osaka, Japan) and 0.0026% hydrogen peroxide (Wako Pure Chemical Industries).

In every PCNA-stained sections obtained per animal, the number of cells with PCNA-positive nuclei (PCNA-positive cells) were counted in the dorsal and ventral areas (each area, 140 × 140 µm) around the ventricle (Fig. 1a). Average dorsal and ventral densities of PCNA-positive cells per animal were calculated, and mean densities and standard errors of PCNA-positive cells were then determined for control (n = 3) and 11-KT injected (n = 3) animal groups.

### Identification of newly-generated cells via *in vivo* BrdU tracing

#### Experimental design for BrdU injections and fixation

Sexually mature female tilapia of almost equal size (n = 6; approximately 30 g in BW and 15 cm length) were used for this study. Three females were simultaneously injected with BrdU (10 mM BrdU (Sigma) at 10 μl/g BW, i.p.) and 11-KT (5 µg/g BW, i.p.), whereas another three were injected with BrdU and the vehicle (sesame oil) for controls. After the injection, the fishes were individually kept in tanks for 7 days. Next, they were anesthetized and transcardially perfused with the fixative as described above. Serial frontal sections were cut at 16 μm thickness on a cryostat. Sections were then thaw-mounted onto MAS-coated glass slides in five parallel series, and stored at −20 °C until further use.

#### Double immunohistochemistry with anti-BrdU and either Hu, GFAP or GnRH3 antibodies

Brain sections obtained as described above were subjected to double immunohistochemistry with anti-BrdU and an antibody against either Hu, GFAP or GnRH3. One series was first stained with anti-GnRH3 to determine the region of the TN where GnRH3-positive neurons were localized. Among the remaining four series of sections, we selected three series of sections, adjacent to the section with the highest number of GnRH3 neurons, to examine the occurrence of newly-generated neurons, GFAP-positive radial glial cells, and GnRH3 neurons. Sections were incubated with anti-Hu C/D (1:100, mouse monoclonal antibody, Molecular Proves, USA), anti-GFAP (1:1000, mouse monoclonal antibody, G3893, Sigma-Aldrich), or anti-GnRH3 antibody. In the next step, sections were incubated in secondary antibodies conjugated with Alexa Flour 555 as follows: goat anti-mouse IgG (1:500, Life Technologies) for Hu C/D (Hu) and GFAP detection, and goat anti-rabbit IgG (1:500, Life Technologies) for GnRH3 detection. For BrdU detection, sections were again fixed in 4% paraformaldehyde and treated with 2N HCl. Immunostaining against BrdU was performed with anti-BrdU antibody (1:400, rat monoclonal antibody, OBT0030, Bio-Rad) and the secondary antibody (Alexa Fluor 488 anti-rat IgG, 1:500, Life Technologies). Stained sections were observed under a confocal laser scanning microscope (LSM5 Pascal, Zeiss). To examine the specificity of the immunoreaction, sections were either processed without the primary antibody, or were incubated with normal rabbit IgG in place of the specific antibody.

### Transcriptome analysis of cellular proliferation-related genes

#### TN sample preparation

Sexually mature female fish of similar size (n = 103, average BW = 28. 6 g and length = 9. 4 cm) were used for this study. Under anesthesia with MS222, 11-KT dissolved in sesame oil was injected intraperitoneally at 5 µg/g BW to nearly half of the fish (n = 53), while the rest (n = 50) were injected with sesame oil. At 24 h after injection, fish were anesthetized and ovarian maturation was confirmed for each fish. From only the matured females (control, n = 40; 11-KT, n = 35), brain regions around the TN were bilaterally removed, weighed (approximately 15 mg/fish), frozen in liquid nitrogen, and stored at −80 °C until use. Next, specimens from three animals were mixed into one sample and RNA extraction was performed with the QIAGEN RNeasy Plus Universal Mini Kit (QIAGEN, Hikden) according to the manufacturer’s protocols. It was found that RNA concentration of each sample, total amount of RNA, RNA integrity number, and 28 S/18 S ratio, were higher than 1 µg/µl, 20 µg, 8.0, and 1.5, respectively. Four control and four 11-KT-treated counterparts were then processed by Genomic BioSci & Tech (New Taipei City, Taiwan) for transcriptome analysis using next generation sequencing (NGS). The rest samples were used for real-time quantitative PCR analysis.

### Library preparation, sequencing and primary analysis

Sampled total RNA were subjected to mRNA isolation and fragmentation, cDNA synthesis, adapter ligation and enrichment amplification. cDNA library was constructed and sequenced using Illumina HiSeq 2000 sequencer (San Diego, CA, USA). CLC genomics workbench (v. 8.0, QIAGEN) was used to conduct various bioinformatics analyses. Readings from Illumina HiSeq sequencing were first trimmed; next, “map read to reference” and “RNA-seq analysis” options were both utilized to map trimmed reads onto *Oreochromis niloticus* genome.

### Differential gene expression cluster analysis

We used gitools v. 2.3 to cluster differentially expressed genes from different treatments. Expression level (RPKM) was calculated using total exon reads/mapped reads (millions) x exon length (kb)). Next, we calculated RPKM Z-score for each gene using Z = (x − μ)/σ, where μ and σ represent the mean and standard deviation, respectively, and used it for cluster analyses with parameters including cluster = 50 and interaction = 300. Subsequently, differentially expressed genes with fold change >1.2, by comparison of RPKM value, were further selected.

### Genes regulating cellular proliferation

Based on the results of our transcriptome analysis, we selected the genes included in the “Cyclins and Cell Cycle Regulation” pathway in PathCards (https://pathcards.genecards.org/Card/cyclins_and_cell_cycle_regulation?queryString=cell%20cycle%20) and “ Cell Cycle” map (map04110) in Kyoto Encyclopedia Genes and Genomes (KEGG) pathway (http://www.genome.jp/dbgetbin/www_bget?pathway:map04110). Identified genes were listed in terms of differential expression between control and 11-KT-treated animals. We used NIH Gene (http://www. ncbi. nlm. nih. gov/gene) to identify the specific function of the genes in the pathways. For the genes whose expression ratios (11-KT treated/control) were greater than 1.2 or less than 1/1.2, we further examined their expression levels by using quantitative real-time RT-PCR analysis.

### Real-time qRT-PCR

The remaining of RNA samples collected for the transcriptome analysis were used. In addition, RNA samples were obtained from the brain regions around the TN of sexually mature female fish at three days after 11-KT injection. Reverse transcription and real-time qRT-PCR were performed as previously reported^63^. The pairs of primers used are listed in Supplementary Table S2.

### Statistical analysis

The numbers of PCNA-, BrdU-, and GnRH3-positive cells, as well as the double-labelled cells (BrdU/Hu, BrdU/GFAP, BrdU/GnRH3) were compared between 11-KT- and control fish using unpaired Student’s *t*-test. Gene expression levels were also compared between these groups using unpaired Student’s *t*-test. *p* < 0.05 was considered statistically significant.

## Electronic supplementary material

Supplementary Dataset (Table S1, S2, S3)
