# Supplementary material for: Androgen induced cellular proliferation, neurogenesis, and generation of GnRH3 neurons in the brain of mature female Mozambique tilapia
Source: Sci Rep. 2018 Nov 15;8:16855. doi: 10.1038/s41598-018-35303-9 (PMC6237963; doi:10.1038/s41598-018-35303-9)
Supplement: Supplementary file 1 — Supplementary Dataset (Table S1, S2, S3) [file 41598_2018_35303_MOESM1_ESM.pdf]

Androgen induced cellular proliferation, neurogenesis, and generation of GnRH3 neurons in the brain of mature female Mozambique tilapia

Yasuto Narita[1], Atsuhiro Tsutiya[1], Yui Nakano[1], Moe Ashitomi[1], Kenjiro Sato[1], Kohei Hosono[2], Toyoji Kaneko[2], Ruo-Dong Chen[3], Jay-Ron Lee[3], Yung-Che Tseng[3], Pung-Pung Hwang[3], Ritsuko Ohtani-Kaneko[1*]

[1]Department of Life Sciences, Toyo University, 1-1-1 Itakura, Oura, Gunma 374-0193, Japan

[2]Department of Aquatic Bioscience, Graduate School of Agricultural and Life Sciences,
The University of Tokyo, 1-1-1 Yayoi, Bunkyo, Tokyo 113-8657, Japan

[3]Institute of Cellular and Organismic Biology, Academia Sinica, Nankang, Taipei City, Taiwan, ROC

* Corresponding author

Ritsuko Ohtani-Kaneko
Department of Life Sciences
Toyo University
1-1-1 Itakura, Oura, Gunma 374-0193, Japan
Phone: +81-276-82-9213; Fax: +81-276-82-9033
E-mail: r-kaneko@toyo. jp

Supplementary Table S1

Gene Symbol		Gene Name	KEGG ID	Path Cards	Raw Deta		Fold Change (11KT/Ctrl)
					Ctrl Means	11KT - Means	
abl1		abelson tyrosine-protein kinase 1 [EC:2.7.10.2]	K06619	-	8.867	9.329	1.052
cdc26	*apc12*	anaphase-promoting complex subunit 12	K03359	-	13.09	10.95	-1.195
apc2		anaphase-promoting complex subunit 2	K03349	-	64.6	63.21	-1.022
cdc27	*apc3*	anaphase-promoting complex subunit 3	K03350	-	51.95	53.15	1.023
cdc16	*apc6*	anaphase-promoting complex subunit 6	K03353	-	20.72	21.5	1.037
cdc23	*apc8*	anaphase-promoting complex subunit 8	K03355	-	13.93	14.88	1.068
dbf4	*ask*	activator of S phase kinase	K06629	-	1.208	1.454	1.203
atm	*tel1*	ataxia telangiectasia mutated family protein [EC:2.7.11.1]	K04728	○	6.911	6.579	-1.05
atr		serine/threonine-protein kinase ATR [EC:2.7.11.1]	K06640	○	2.579	2.626	1.018
btg1		BTG anti-proliferation factor 1	-	○	52.64	54.94	1.043
bub1		checkpoint serine/threonine-protein kinase [EC:2.7.11.1]	K02178	-	0.8691	0.793	-1.095
bub1b	*bubr1,mad3l*	mitotic checkpoint serine/threonine-protein kinase BUB1 beta [EC:2.7.11.1]	K06637	-	1.708	1.753	1.026
bub3		cell cycle arrest protein BUB3	K02180	-	23.49	24.87	1.058
ccna1		cyclin A1	-	○	0.607	0.4547	-1.334
ccna2		cyclin A2	-	○	1.956	1.687	-1.159
ccnb1		G2/mitotic-specific cyclin-B1	K05868	○	1.859	1.52	-1.223
ccnb2		cyclin B2	-	○	0.2677	0.4126	1.541
ccnb3		G2/mitotic-specific cyclin-B3	K21771	○	0.8934	1.417	1.586
ccnd1		G1/S-specific cyclin-D1	K04503	○	6.349	6.433	1.013
ccnd2		G1/S-specific cyclin-D2	-	○	0.08642	0.04453	-1.94
ccnd3		G1/S-specific cyclin-D3	K10152	○	0	0	1
ccne1		cyclin E1	-	○	8.04	8.104	1.008
ccne2		G1/S-specific cyclin-E2	-	○	0.4203	0.6588	1.567
ccnh		cyclin H	K06634	○	20.44	21.15	1.034
cdc20		cell division cycle 20, cofactor of APC complex	K03363	-	2.997	2.521	-1.188
cdc25Bb		M-phase inducer phosphatase 2 [EC:3.1.3.48]	K05866	-	4.68	5.271	1.126
cdc45		cell division control protein 45	K06628	-	3.349	3.886	1.16
cdc6		cell division control protein 6	K02213	-	0.8925	0.31	-2.878
cdc7		cell division control protein 7 [EC:2.7.11.1]	K02214	-	1.264	1.67	1.321
cdk1	*cdc2*	cyclin-dependent kinase 1 [EC:2.7.11.22 2.7.11.23]	K02087	○	5.112	6.038	1.181
cdk2		cyclin-dependent kinase 2 [EC:2.7.11.22]	K02206	○	6.522	6.648	1.019
cdk4		cyclin-dependent kinase 4 [EC:2.7.11.22]	K02089	○	6.825	7.88	1.154
cdk6		cyclin-dependent kinase 6 [EC:2.7.11.22]	K02091	○	10.04	10.34	1.029
cdk7		cyclin-dependent kinase 7 [EC:2.7.11.22 2.7.11.23]	K02202	○	28.96	28.54	-1.014
cdkn1c	*p57,kip2*	cyclin-dependent kinase inhibitor 1C	K09993	○	0.2757	0.2416	-1.14
cdkn2c	*p18,ink4c*	cyclin-dependent kinase inhibitor 2C	K06622	○	0.08689	0	-1000
cdkn2d	*p19,ink4d*	cyclin-dependent kinase inhibitor 2D	K06623	○	16.89	17.23	1.02
cks1b		CDC28 protein kinase regulatory subunit 1B	-	○	2.291	2.828	1.234

cnot7		CCR4-NOT transcription complex subunit 7	-	○	20.79	19.19	-1.083
cul1	*cdc53*	cullin 1	K03347	○	126.9	127.5	1.004
cyce3		G1/S-specific cyclin-E3 (Xenopus laevis)	-	-	0.09458	0.02204	-4.291
e2f1		transcription factor E2F1	K17454	○	2.562	2.136	-1.199
e2f2		transcription factor E2F2	K09389	○	0.4275	0.6081	1.422
e2f3		transcription factor E2F3	K06620	○	5.59	5.495	-1.017
e2f4		transcription factor E2F4	-	○	16.68	17.54	1.051
crebbp	*ep300,kat3*	E1A/CREB-binding protein [EC:2.3.1.48]	K04498	-	23.01	23.53	1.022
gsk3b		glycogen synthase kinase 3 beta [EC:2.7.11.26]	K03083	○	50.98	50.44	-1.01
hdac10		histone deacetylase 10	-	○	8.703	8.437	-1.031
hdac11		histone deacetylase 11	-	○	37.58	37.79	1.005
hdac2		histone deacetylase 2	-	○	15.64	14.31	-1.092
hdac3		histone deacetylase 3	-	○	37.9	39.18	1.033
hdac5		histone deacetylase 5	-	○	34.11	33.15	-1.028
hdac6		histone deacetylase 6	-	○	12.82	13.78	1.074
hdac8		histone deacetylase 8	-	○	10.44	10.9	1.043
hdac9		histone deacetylase 9	-	○	85.97	89.58	1.042
hoxb9		homeobox B9	-	○	0	0	1
mad2l2		mitotic spindle assembly checkpoint protein MAD2B	K13728	-	21.23	19.28	-1.101
mcm2		DNA replication licensing factor MCM2 [EC:3.6.4.12]	K02540	-	4.046	4.183	1.033
mcm3		DNA replication licensing factor MCM3 [EC:3.6.4.12]	K02541	-	10.25	9.704	-1.056
mcm4	*cdc54*	DNA replication licensing factor MCM4 [EC:3.6.4.12]	K02212	-	18.94	19.82	1.046
mcm5	*cdc46*	DNA replication licensing factor MCM5 [EC:3.6.4.12]	K02209	-	2.725	2.878	1.056
mcm6		DNA replication licensing factor MCM6 [EC:3.6.4.12]	K02542	-	3.702	4.06	1.096
mcm7	*cdc47*	DNA replication licensing factor MCM7 [EC:3.6.4.12]	K02210	-	11.28	12.04	1.067
mdm2		E3 ubiquitin-protein ligase Mdm2 [EC:2.3.2.27]	K06643	-	13.28	13.9	1.046
mnat1		MNAT1, CDK activating kinase assembly factor	-	○	15.57	15.68	1.007
myc		Myc proto-oncogene protein	K04377	-	18.43	18.39	-1.001
noct		nocturnin	-	○	6.568	6.84	1.041
orc1		origin recognition complex subunit 1	K02603	-	1.789	1.913	1.069
orc2		origin recognition complex subunit 2	K02604	-	2.707	2.553	-1.06
orc3		origin recognition complex subunit 3	K02605	-	8.229	6.796	-1.21
orc4		origin recognition complex subunit 4	K02606	-	3.279	3.54	1.079
orc5		origin recognition complex subunit 5	K02607	-	6.843	6.727	-1.017
pcna		proliferating cell nuclear antigen	K04802	-	51.51	52.44	1.018
plk1		polo-like kinase 1 [EC:2.7.11.21]	K06631	-	2.339	2.234	-1.047
ppp2ca		protein phosphatase 2 catalytic subunit alpha	-	○	127.2	129.3	1.016
ppp2r2c		protein phosphatase 2 regulatory subunit Bgamma	-	○	116.8	114.7	-1.017
ppp2r3a		protein phosphatase 2 regulatory subunit B''alpha	-	○	14.77	15.01	1.015
ppp2r5a		protein phosphatase 2 regulatory subunit B'alpha	-	○	27.96	28.53	1.02
ppp2r5b		protein phosphatase 2 regulatory subunit B'beta	-	○	186.6	186.1	-1.002

ppp2r5c		protein phosphatase 2 regulatory subunit B'gamma	-	○	52.02	41.39	-1.257	
ppp2r5e		protein phosphatase 2 regulatory subunit B'epsilon	-	○	60	59.77	-1.003	
prkdc		DNA-dependent protein kinase catalytic subunit [EC:2.7.11.1]	K06642	-	6.434	5.952	-1.08	
prmt1		protein arginine methyltransferase 1	-	○	283.6	272.5	-1.04	
raf1		Raf-1 proto-oncogene, serine/threonine kinase	-	○	14.62	15.47	1.058	
rb1		retinoblastoma-associated protein	K06618	○	15.29	13.91	-1.099	
rbl1		retinoblastoma-like protein 1	K04681	○	4.399	4.176	-1.053	
rbl2		retinoblastoma-like protein 2	K16332	○	1.347	1.972	1.464	
rbx1	*roc1*	RING-box protein 1	K03868	-	103.3	106.3	1.028	
rps27a		ribosomal protein S27a	-	○	2409	2521	1.046	
rad21	*scc1,mcd1*	cohesin complex subunit SCC1	K06670	-	0	0	-1000	
skp1	*cbf3d*	S-phase kinase-associated protein 1	K03094	○	424.1	415.7	-1.02	
skp2	*fbxl1*	F-box and leucine-rich repeat protein 1 (S-phase kinase-associated protein 2)	K03875	○	0.8477	0.5443	-1.557	
smc3	*cspg6*	structural maintenance of chromosome 3 (chondroitin sulfate proteoglycan 6)	K06669	-	114.6	113.8	-1.006	
tfdp1		transcription factor Dp-1	K04683	○	18.56	18.63	1.003	
tgfb2		transforming growth factor beta-2	K13376	○	11.05	11.19	1.012	
tgfb3		transforming growth factor beta-3	K13377	○	7.899	8.394	1.062	
p53	*tp53*	tumor protein p53	K04451	○	36.35	37.01	1.018	
ttk	*mps1*	serine/threonine-protein kinase TTK/MPS1 [EC:2.7.12.1]	K08866	-	1.656	1.892	1.142	
uba52		ubiquitin A-52 residue ribosomal protein fusion product 1	-	○	864.5	913.8	1.057	
ubb		ubiquitin B	-	○	3733	3936	1.054	
ubc		polyubiquitin	-	○	0.4537	5.068	11.16	
wee1		wee1-like protein kinase [EC:2.7.11.1]	K06632	○	2.433	2.654	1.091	
zbtb17	*miz1*	zinc finger and BTB domain-containing protein 17	K10500	-	15.5	14.97	-1.035	

Supplementary Table S2. Primer sequences for real-time RT-PCR.

Ccna1	Fw	ACACCCAGAGATCACGGATG
	Rv	TGAGGGAGAGAAATCGGTCCA
Ccnb1	Fw	GCCTACACCACAGCCCAAAT
	Rv	CCCTTCTGAGGAACTGCAGG
Ccnb2	Fw	AACCTGGAGTTGCAGCAGAC
	Rv	GACCAGCCAGTCGATGAGAA
Ccnb3	Fw	CTGGCTGGTCGAAGTACAGG
	Rv	GACCGGGGTTTTAGCCAAGT
Ccnd2	Fw	GTGGTGTGAGGAAGTGGAGG
	Rv	CTCTGGATCACTCGATGCCC
Ccne2	Fw	GGAGTGAACTTCCTGGAGCC
	Rv	GTTATAGTCGAGCGCGGTGA
Rbl2	Fw	CCCCAACACACCATCAACAC
	Rv	GACAAAGGAGGGGTGTCTCG
E2f2	Fw	GGCCAAGAGGAAGCTGGATT
	Rv	GTATCCGTGCTGCAATGCTG
Cks1b	Fw	CAAGCGTGTTCCCAAAACCC
	Rv	TGCACCCATCCTTGACTCTG
Cdc6	Fw	GGACTGCAGAGGTTCCTGAC
	Rv	CGATGAGACAGAGGCGAGAC
Cdc7	Fw	AAGTCGTTCGGTAAAGCGGT
	Rv	CCTCTGAGCGTCTCACACAG
Skp2	Fw	GCGTTTGACGAGTCTCTGTG
	Rv	CTGCGGATGCCAGTCCTCA
Cyce3	Fw	CGCTGTTCACAGAGAGACGT
	Rv	GCAGCACACCCAACTTAAGG
Cdkn2c	Fw	AAGCGGGGGCAAATCCAAAT
	Rv	CGCACAGAATCCACGAATCC
Dbf4	Fw	ACAAACAGCGGGGACAAAGA
	Rv	ATACAGCACTCGCAGTAGCC
Ubc	Fw	CTGGTTTTCATAAACGGCCAGA
	Rv	GAGGCTCGGTGACCAGCA
Ppp2r5c	Fw	TGGAAGGAGGTGAAACGAGC
	Rv	TCTGGGTAGATCGGTTCGGT
Orc3	Fw	GCACTGCTCTCTCGGGATAC
	Rv	TGCTGCTGCTCATCTCTGAC
Rad21	Fw	AACAACAGGCCACCAACAGA
	Rv	CGACTCATCCAGGGTTGTCC
Ara	Fw	CAGAGGGGAGAATCAGGGGA
	Rv	CGTACCAGATACAGCGGCAT

Arb	Fw	GTACGAGCACTGCATACGGA
	Rv	TGCTAAAGAGGAGCAAGGCC
Era	Fw	GGCTTGCCGTCTTAGGAAGT
	Rv	GCAGGTCTTTGGCTGGTTTG
Erb	Fw	GGGTCTCACTTGGACAGCAA
	Rv	TGACGTAGGTTGTTGGCTCC
Gnrh3	Fw	AATGCAGGTGTTGTTGCTGG
	Rv	GGTCAAAGTGACTGGAATCATCA

Supplementary Table S3

Gene Symbol	Gene Name	KEGG ID	Transcriptome analysis		
			Raw data (Cont)	Raw data (11KT)	Fold change
Arα	andorogen receptor alpha	K08557	4.221	3.846	-1.097
Arβ	androgen receptor	K08557	1.956	2.615	1.337
Erα	type I estrogen receptor	K08550	27.05	27.53	1.017
Erβ	estrogen receptor 2	K08551	1.505	1.776	1.180
Gnrh3	progonadoliberin-3	K05252	116.4	132.2	1.135
